# Modelling the distribution of the tick *Ixodes ricinus* in England and Wales using passive surveillance data from citizen science reports

**DOI:** 10.1371/journal.pntd.0013520

**Published:** 2025-10-09

**Authors:** Mark Gideon Burdon, Maximilian Ayling, Nyall Jamieson, Julie Day, Jolyon Medlock, Kayleigh Hansford, G. R. William Wint, Thomas Ward

**Affiliations:** 1 UK Health Security Agency, London, United Kingdom; 2 Department of Mathematics, University of Manchester, Manchester, United Kingdom; 3 Department of Biology, University of Oxford, Oxford, United Kingdom; Creighton University, UNITED STATES OF AMERICA

## Abstract

**Background:**

Ticks are a significant cause of illness globally. The tick *Ixodes ricinus* is commonly found across Europe and is a significant vector of Tick-Borne Encephalitis virus (TBEv), *Borrelia burgdorferi* s.l. (causative agent of Lyme borreliosis), *Babesia divergens*, *Anaplasma phagocytophilum*, and several *Rickettsia* bacteria, among others.

**Methods:**

The Tick Surveillance Scheme (TSS) administered by the UK Health Security Agency (UKHSA) contains validated reports from the general public of tick encounters over the last twenty years. We modelled the probability of *I. ricinus* tick presence across England and Wales using the locations of TSS reports from 2013 to 2023 and a combination of biotic and abiotic factors. An ensemble of statistical and machine learning models was trained to classify points as presence (true tick report locations) or background (points generated randomly and by target-group sampling).

**Results:**

The ensemble model had a continuous Boyce index of 0.99 and area under the receiver-operator curve (ROC AUC) of 0.84 on out-of-sample 2024 data. Variables relating to roe deer (*Capreolus capreolus*) distribution and land cover type were most important. Most of southern England, as well as other areas with known tick populations such as the New Forest and the Lake District, are modelled as highly probable tick presence areas.

**Interpretation:**

Unstructured citizen science data was suitable for creating a high-performing species distribution model for *I. ricinus* after addressing spatial and demographic biases. This model is now being used to inform local public health awareness showing the advantage of passive surveillance through to modelling and public health awareness.

## Author summary

Ticks are concerning for public health because their bite can pass on a wide range of diseases to humans. Understanding which areas have ticks allows local public health agencies to target their work more effectively, but manually searching for ticks takes considerable time and resources. One alternative is to record where ticks have been found by members of the public (including vets, health professionals and scientists), ensuring that the tick specimens are collected and verified. We collected locations of verified deer tick encounters in England and Wales from the UK Health Security Agency’s Tick Surveillance Scheme. We then collated information about the environment surrounding the tick locations, including average temperatures, whether deer are thought to be present, how much of the area is woodland, etc. This allowed us to create models that estimate how likely each part of England and Wales is to have ticks and create a detailed risk map. The main predicted area for the deer tick was in southern England, while the Midlands and much of Wales were less likely to have ticks; this generally agrees with previous research. On average, the model classified areas with deer and more woodland as more likely to have ticks.

## Introduction

Ticks play a significant role in the transmission of zoonotic disease globally, transmitting a range of pathogenic micro-organisms - including protozoa, rickettsiae, spirochaetes and viruses - to humans [1]. Novel tick-borne pathogens continue to emerge in the 21st century, including the Heartland and Bourbon viruses in 2009 and 2014 [2]. Understanding the distribution and spread of ticks is therefore important for public health globally.

The tick *Ixodes ricinus* is commonly found across Europe and is a significant vector of Tick-Borne Encephalitis virus (TBEv), *Borrelia burgdorferi* s.l. (causative agent of Lyme borreliosis), *Babesia divergens*, *Anaplasma phagocytophilum*, and several *Rickettsia* species, among others. In the UK, *I. ricinus* has been confirmed as a vector of TBEv since 2019, with now a small number of probable and confirmed cases recorded having been acquired locally [3]. From a veterinary perspective, *I. ricinus* is a known vector of louping ill virus [4], tick-borne fever (caused by *Anaplasma phagocytophilum*) [5] and bovine babesiosis (caused by *Babesia divergens*) [6].

*Ixodes ricinus* ticks appear to have also spread into new areas of England since the early 2000s [7,8]. A central component to understanding and mitigating the risk ticks pose to human health is a reliable understanding of where tick populations are likely to be found and where humans might come into contact with them. Targeted field surveillance for ticks carried out by scientifically trained professionals can be resource intensive and therefore have limited spatial and temporal coverage [9,10]. Citizen science projects to record species observations are therefore highly valuable for use in passive surveillance of infectious disease vectors (see also [11]), which can be enhanced when combined with expert verification and validation processes. The TSS identification validation process is critical to prevent morphologically similar species (for example *Ixodes hexagonus*), with varying ecologies, being misclassified. The ability to estimate spatial vector distributions from unstructured citizen science data is important for public health and, at the time of writing, no detailed tick risk map for England and Wales was accessible online.

This study will be of interest to public health agencies globally who aim to use passively collected citizen science data to model vector species distributions. It models the spatial distribution of *I. ricinus* across England and Wales using the Tick Surveillance Scheme (TSS) data collected by the Medical Entomology group in the UK Health Security Agency (UKHSA, previously in Public Health England and Health Protection Agency). The TSS contains expert validated reports of tick encounters sent in by human and animal health professionals and the public (with tick submissions after a bite of a human or animal). Submissions must include a live or dead tick specimen, and include a form that provides details of how and where the tick was encountered. UKHSA’s Medical Entomology and Zoonoses Ecology team identify each specimen and record geographic coordinates for the likely acquisition location, noting how these were derived.

## Methodology

### Data

#### Vector presences.

We used 4,083 TSS records from England and Wales that were verified by UKHSA as *I. ricinus* from between 2013 and 2023 inclusive, with partial records for 2024 held back for use only in model testing to avoid spatial autocorrelation resulting in inflated performance statistics [12]. With most of the reports in England and Wales, data from Scotland and Northern Ireland was excluded from the study. Duplicate reports from the same recorder in the same location and year were also excluded, as this study is focused on modelling presence.

The TSS is based on passive surveillance data from citizen science reports, which introduces spatial biases. There is heterogeneous sampling effort across the study area due to differences in where individuals with a higher propensity to submit reports live or visit - such as urban areas or popular nature parks, as opposed to inaccessible private woodland or farmland [13,14]. Because this pro-urban spatial heterogeneity was apparent in the raw data, we addressed it by a) varying importance weights for presences based on population density at the tick report location; and b) using target-group sampling to generate background points.

**Fig 1:**
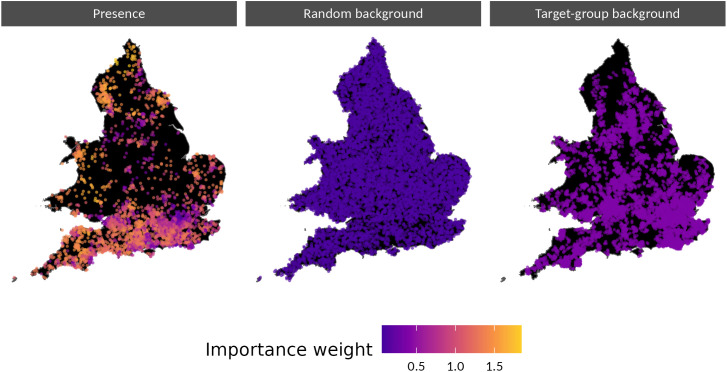
Maps showing the locations and relative weights given to presence points, randomly generated background points and target-group sampled background points. Areas with no points are filled in black. In the plot, all point locations are jittered for pseudonymisation. Country boundaries source: Office for National Statistics licensed under the Open Government Licence v.3.0.

To reduce the spatial bias arising from higher observer presence in populous areas, we used importance weights that assigned more influence to occurrences in sparsely populated areas, using normalised log population density of the tick location’s 2021 Middle layer Super Output Area [15]. Weighting for sampling effort has previously been shown to increase accuracy in modelling bird presence [16]. The differences in these weights can be seen graphically in the presences subplot of Fig 1.

#### Background points.

Previous modelling research has noted that obtaining true absence data for ticks is challenging because a species’ niche changes over time [17] and with weather conditions [18] and seasonality, and sampling via dragging is resource-intensive [10]. Standard practice is to generate either “pseudo-absence” points, often stratified by how confident the researcher is of absence, or “background” points that represent the overall environmental space, including areas where the species may be present. However, neither of these methods addresses the issue of spatial bias due to differential sampling effort; therefore, we combined random background point generation with target-group background sampling. Target-group sampled background points are generated based on presence reports of other species in the same taxonomic group as the one targeted by the SDM and has been shown to be effective at reducing spatial bias [19,20]. Species of the same taxonomic group are likely to have similar detectability and sample biases. We anticipate this may also reduce biases arising from differential propensity to report ticks among different human demographics. We generated two target-group sampled background points for each presence point, distributed according to the density of other tick species reports from the TSS. A 2:1 ratio was selected because of recommendations from Liu et al. (2019) to create background points as a small multiple of the total presences.

However, iterative model development showed that relying entirely on target-group sampling resulted in a lack of background points in some areas (e.g., the north Pennines) that had very few TSS reports for any species of tick. To address these gaps, geographically random background points were also generated within the boundaries of England and Wales, with a minimum distance of 5km from presences (to reduce overlap between presence and background points). Two randomly generated points were created for each presence point, resulting in a total of four background points per presence point.

The effect of this combination is that background points were distributed throughout the geographical and environmental space of England and Wales, but are more likely to be produced in areas where other ticks have been reported. To increase the influence of the target-group sampled background points, these were assigned a weight of 0.8 (compared to 0.2 for randomly generated points). The combined target-group and random background points were then re-weighted such that the total weight of all background points was approximately equal to the total weight of all presence points, following the finding from [21] that a combination of low background-to-presence ratio and equal total group weight between presences and background points performs well in SDMs.

A version of the model without randomly generated background points, and a version with more background points, are demonstrated in the Sensitivity Testing section, as are alternative weighting schemas (e.g., placing more emphasis on target-group sampled points) and a version of the model without buffers around the randomly generated background points. Fig 1 shows the location of both presence and background points in the training set and their relative weighting; Fig A in S1 Text compares the total weight assigned to each type of point (presence, target-group sampled background, random background).

#### Environmental data.

A set of environmental predictor variables, which have previously been associated with the presence of *I. ricinus*, were identified and collated from publicly available data sources. These predictor variables fall into the following categories: climatic conditions, land cover, soil type, geology, host prevalence (cattle, pig, sheep and six species of deer), NDVI and elevation.

Although the *I. ricinus* tick can only crawl a few metres [22], it has a wide range of hosts including deer, livestock, rodents, birds and small mammals, all of which can help with tick dispersal and it can adapt to live in a range of habitats (e.g., [deciduous, coniferous, mixed] woodland, woodland edge, moorland, heathland, grazed grassland, urban parks) provided there is a suitable microclimate to support off-host survival [23]. As a result, many different factors may contribute in complex ways to determine whether if the species is introduced to an area, and if it is able to thrive in that locality. The increase in deer numbers and their expanding range across the UK, along with as land-use changes and climactic variation, may have contributed to an enlarged geographic range for *I. ricinus* [8,24,25]. We therefore aimed to include a diverse range of factors that have some *a priori* ecological justification as to their impact on *I. ricinus*, and are not confounded with spatial bias.

Existing modelling indicates that temperature has a non-linear effect on *I. ricinus* presence and abundance [7]. We therefore include data on temperature ranges, sunshine hours, ground frost and snow lying days from HadUK-Grid 1km v1.3.0 [26]. HadUK-Grid rainfall and humidity were also extracted as ticks are more prevalent in humid environments and risk desiccation in drier climates [27], though we recognise that there are concerns about the relevance of macroclimatic humidity to the microclimate experienced by ticks [10,28]. Soil type has been shown to be predictive of tick presence [29,30]; we included WRBLV1 from the European Soil Data Centre’s European Soil Database v2.0 [31,32]. We used land cover type from the UKCEH Land Cover Map for 2021 [33] as we expect woodland and scrubby, grazed grassland areas to be high-risk areas for tick presence [10,34–37]; and to give a broader view of the likely ecology and biodiversity of the area, we also use superficial deposit type from the British Geological Survey [38], as superficial geology and soil permeability has been linked to tick presence [39]. NDVI is often important in predicting *I. ricinus* presence and abundance [10,40–43]; the median NDVI for each pixel is calculated across April to August in each year after masking the “cloud” and “cloud shadow” layers. This time period was chosen as the peak in TSS reporting activity [36], and in line with previous SDMs’ exclusion of winter NDVI [42]. NDVI was calculated based on satellite images from USGS Landsat 8 Level 2, Collection 2, Tier 2, accessed via the *rgee* interface to Google Earth Engine [44]. Modelled estimates for presence of and environmental suitability for six deer species (fallow, roe, red, Chinese water deer, Chinese muntjac and Japanese sika) were included [45], as were estimated densities for cattle [46], pigs [47] and sheep [48]. Finally, because the absence of hosts at high altitudes can affect tick distribution [7], we also include elevation from the NASA Shuttle Radar Topography Mission (SRTM) digital elevation data [49].

To supply the model with an approximate picture of the composition of the area around the tick report (for example 56% built-up areas and gardens, 28% arable, etc.), categorical variables were calculated as proportions of a 1km-wide grid square. Each tick report or background point was therefore linked to the environmental conditions of the grid square in which it fell. All spatial data was re-projected to 1km x 1km resolution and cropped to the extent of England and Wales. To reduce inference problems caused by high collinearity between covariates, pairs of variables with the highest Pearson correlation above 0.7 were identified, and one variable was removed from each pair using the *step_corr* function from the *recipes* R package [50].

### Models

A set of four base SDMs (two statistical models and two machine learning models) were trained on the 2013–2023 training data to distinguish *I. ricinus* presences from background points, using spatial block cross-validation from the *tidysdm* R package [51]. Twelve potential hyperparameter combinations for each model were tuned using a grid search with racing [52]. For each of the four model types, the hyperparameter combination with the highest average continuous Boyce index was then included in the ensemble. The ensemble model took the average of the predicted presence probability for each data point from the four finalised base models.

#### Statistical models.

A penalised generalised linear model (pGLM) and a generalised additive model (GAM) were estimated using the *parsnip* package as part of a broader *tidymodels*-based workflow.

The pGLM was specified as a penalised logistic regression model with alpha and lambda terms selected by cross-validation. After applying regularisation and penalisation, the model equation is that of a standard logistic regression model:


$$\begin{array}{c} \text{logit}(p)=\text{log}\left(\frac{p\left(\text{Presence}\right)}{\text{1}-p\left(\text{Presence}\right)}\right)=\beta_{\text{0}}+\beta_{\text{1}}{\text{groundfrost}}_{\text{min}}+\beta_{\text{2}}{\text{groundfrost}}_{\text{max}}+\beta_{\text{3}}{\text{rainfall}}_{\text{min}}+\ldots +\beta_{p}x_{p}. \end{array}$$

The Generalised Additive Model (GAM) was specified as a binomial-family GAM. The equation that defined the GAM was written as follows:


$$\begin{array}{cc} \text{logit}(p)=\text{log}\left(\frac{p(Presence)}{\text{1}-p(Presence)}\right)= & \;\beta_{\text{0}}+s_{\text{1}}\left(\text{groundfrost}\;\text{max},\text{sun}\;\text{min},\text{sun}\;\text{max},\text{rainfall}\;\text{mean}\right)\\ & +s_{\text{2}}(\text{coniferous}\;\text{woodland},\;\text{broadleaf}\;\text{woodland},\\ & \text{built}\;\text{up}\;\text{areas}\;\text{and}\;\text{gardens},\;\text{arable})\\ & +s_{\text{3}}\left(\text{mountain},\;\text{heath}\;\text{and}\;\text{bog}\right)\\ & +s_{\text{4}}(\text{red}\;\text{suitability},\;\text{red}\;\text{current}\;\text{distribution},\;\text{roe}\;\text{current}\;\text{distribution},\;\\ & \text{Chinese}\;\text{water}\;\text{deer}\;\text{current}\;\text{distribution})\\ & +s_{\text{5}}\left(\text{sheep}\;\text{population}\right) \end{array}$$

where


i∈{1,2,3,4,5}

were thin-plate regression splines.

Key variables were combined thematically into splines (weather, land cover type, deer presence and suitability, and sheep) to allow the model to use combinations of conditions (e.g., areas with a mix of forest and grassland habitats). “Mountain, heath and bog” was estimated in its own spline due to cross-validation issues related to its sparsity. The number of knots in each spline was determined iteratively to maximise model accuracy. Splines $s_{\text{1}}$, $s_{\text{2}}$ and $s_{\text{3}}$ had five knots, $s_{\text{4}}$ had seven and $s_{\text{5}}$ had six knots.

#### Machine learning models.

An extreme gradient boosted trees (XGBoost) model from the *xgboost* package [53] and a Random Forests model from the *ranger* R package [54] were estimated in classifier mode via *parsnip* with early stopping. Details of model hyperparameters are shown in S1 Text.

## Results

### Model performance

**Fig 2:**
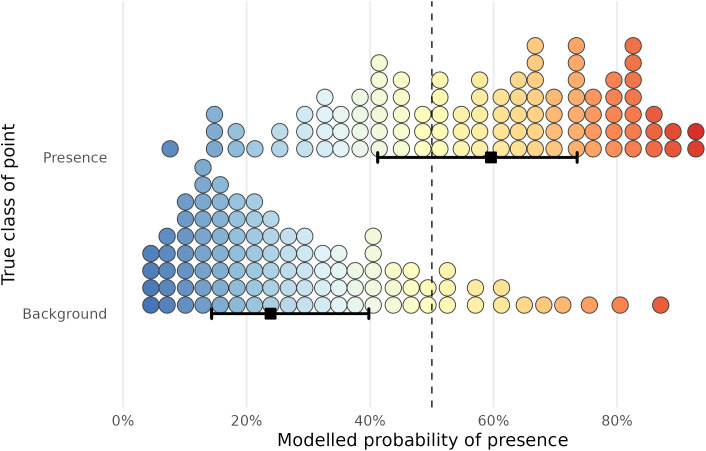
Wilkinson dot plots and intervals showing the distribution of the probability assigned to points in the 2024 testing data by the ensemble model, split by class (whether the point was a true tick presence point or a background point). Each bin represents 1% of the distribution, and each bin represents an equal number of observations (Kay 2023). The square point shows the median, and the thick black horizontal line shows the central two quartiles of the distribution. The dashed vertical line represents the 50% threshold.

Predictions on the 2024 testing set were compared with the true class to assess the ensemble model’s ability to distinguish risk of *I. ricinus* presence. As climate data for 2024 were not available at the time of analysis, these variables were set as their mean values for the 2021–2023 period, in effect creating a naive forecast for 2024. Performance metrics were calculated on unweighted counts. 63% of presences and 86% of background points were correctly classified. Fig 2 shows the modelled probabilities that the ensemble model assigned to the 2024 testing data.

The median prediction for true presence points was 60%, and for background points was 24%. This indicates that the model was more skilled at classifying background points than presence points in the 2024 testing data. Table 1 shows a range of model performance metrics for the simple ensemble model and the four base models.

**Table 1:** Out-of-sample predictive performance metrics for the simple ensemble and base models.

Metric	Simple ensemble	pGLM	GAM	XGBoost	Random Forest
Accuracy	0.82	0.71	0.72	0.83	0.84
Kappa	0.47	0.29	0.28	0.48	0.50
MCC	0.47	0.31	0.30	0.48	0.50
Sensitivity	0.63	0.63	0.58	0.62	0.58
Specificity	0.86	0.73	0.76	0.88	0.91
Boyce Continuous	0.99	0.99	0.97	0.98	0.99
ROC AUC	0.84	0.76	0.74	0.85	0.87
Brier score	0.14	0.19	0.19	0.12	0.11
Max TSS	0.53	0.41	0.39	0.56	0.57

The machine learning-based models (XGBoost and Random Forest) generally scored higher on measures of overall performance. The statistical models’ sensitivity (ability to correctly detect presence points) was very similar to the machine learning models, but their specificity was lower (they incorrectly assigned more background points as presences). The simple ensemble’s scores on these metrics were closer to those of the machine learning models than the statistical models, and in some cases were equal to the XGBoost model.

To check how sensitive the model’s outputs are to different researcher choices, we carried out a range of sensitivity tests, each time making only one change from the baseline specification described in the main body of the paper. These are described in S1 Text.

### Prediction maps

**Fig 3:**
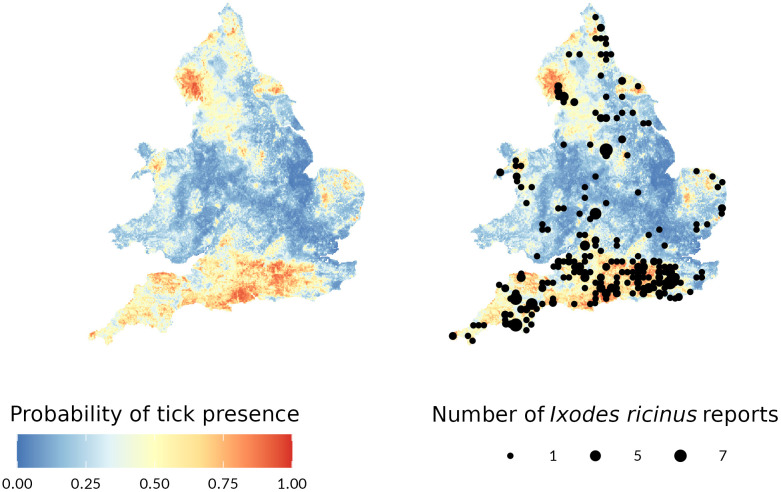
Modelled presence probabilities and actual tick reports from the 2024 testing data. To prevent reidentification of exact locations, reports were aggregated in a 10km x 10km grid and are shown at the centroid of the grid square. Larger dots represent multiple reports in the same grid square. Country boundaries source: Office for National Statistics licensed under the Open Government Licence v.3.0.

As well as statistical model performance assessment, it is important to sense-check model predictions against expert opinion and other available evidence. Fig 3 shows the modelled presence probabilities and out-of-sample TSS tick report locations for 2024.

Areas with high modelled probability of *I. ricinus* presence include parts of northern England including notably Cumbria and the North Yorkshire Moors. In Wales, the highest modelled probabilities are in parts of Eryri in the north-west. In the Midlands and Anglia, there is currently lower suitability for *I. ricinus*, especially in Lincolnshire, largely on account of lower deer densities, although areas such as Thetford Forest and the Cotswolds are exceptions. The main predicted area for *I. ricinus* is in southern England, particularly Dorset, the New Forest, the South Downs, Exmoor and Dartmoor. The only exception is parts of east Kent; however, this may change as deer populations spread to occupy this area.

The map was inspected by UKHSA medical entomology co-authors for expert review, checking the maps aligned with the understanding of *I. ricinus* distribution. In general, the maps were in line with expectations. Two areas where the prediction maps did not align with the co-authors’ understanding of the distribution were in the sheep farming areas of North Wales where ticks are expected to be more prevalent than the maps suggest; conversely, in the sheep-grazed uplands of the Lake District, there is anecdotally little evidence of ticks at high altitudes (as opposed to the forested valleys).

### Variable importance and interpretability

**Fig 4:**
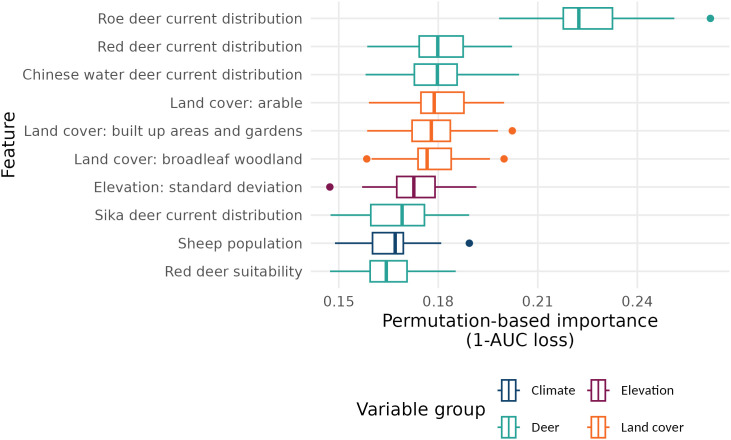
Permutation-based variable importance for simple ensemble predictions on the testing set. The plot shows the ten most important variables on average across 25 iterations, along with confidence intervals. Loss is measured as impact on 1-ROC AUC.

We used post-hoc explainability techniques to allow some non-causal interpretation of machine learning model predictions [55]. Variable importance from model-agnostic permutation methods [56,57] from the *DALEX* R package [58] were used to produce Fig 4, which shows the ten most important variables across 25 permutation iterations.

**Fig 5:**
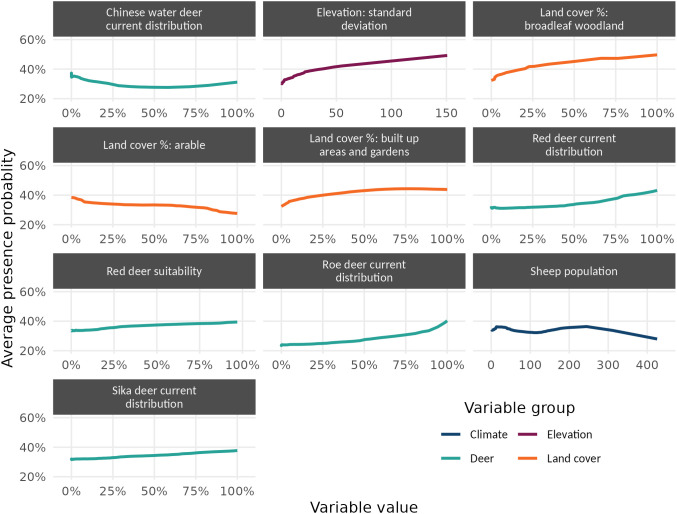
Partial dependence plots (PDPs) showing the average presence probability from the simple ensemble model, for the full range of the ten most important predictors in the testing data. All other predictors are held at their mean in each plot.

Partial dependence plots (PDP) show how average predictions vary across the range of a predictor when all other predictors are held at their mean. Fig 5 contains a PDP for each of the ten most important variables.

## Discussion

This paper produces, to our knowledge, the first published species distribution model for *I. ricinus* in England and Wales at the 1km resolution or similar. Previously published risk maps for *I. ricinus* in England have been modelled at a coarser resolution than this study [59,60], making them unsuitable for local health interventions. Other UK studies investigating the risk of tick-borne diseases have focused on particular locations within the country, such as national parks [61] and urban green spaces [62], both of which were focused on the variability of infection rates and the drivers for risk. We were interested in determining whether passive surveillance datasets, such as the UKHSA Tick Surveillance Scheme [36], are suitable for species distribution modelling, and what steps public health agencies can take to improve the reliability of models based on these data collection schemes. To enable greater reproducibility, we used open-source software, and in particular chose to use software packages that are compatible with the general purpose *tidymodels* modelling framework [51].

We found that applying less weight to presence data from densely populated areas and using target-group background sampling was effective in reducing presence predictions in towns and cities, and we expect this to also apply to other human biases introduced by the citizen science sampling process. Using an ensemble of statistical and machine learning models resulted in a ‘risk map’ showing the modelled probability of tick presence that was consistent with expert knowledge of *I. ricinus* ecology and distribution, as well as with previous small-scale survey-based models. Consistent with the literature, areas with broadleaf woodland and deer presence were modelled as higher risk for *I. ricinus*. Some areas with moderately high presence probabilities may be as yet unrealised parts of the species’ environmental niche. Further work is needed to validate or challenge predictions in areas such as North Wales and the Lake District where some granular predictions did not match expectations based on the ecology. In the absence of an alternative hypothesis to explain the discrepancy in North Wales, this may arise due to differential awareness of the scheme and therefore lower propensity to report ticks in the area. The Lake District issue may arise due to there being sharp differences in habitat between the valleys and peaks in the Lake District that are effectively smoothed over by the model’s grid system; in other words, within the same square kilometre you may have a suitable habitat (forested valley) and an unsuitable peak. The model may then have extrapolated that areas with highly variable elevation (rugged landscapes) are suitable habitats for *Ixodes ricinus* due to ticks picked up in the forested areas. In addition, the use of population density as a proxy for sampling effort is likely to understate the amount of outdoor activity undertaken in this region, and therefore the propensity for ticks to be found and reported.

However, we recognise that our modelling approach has limitations and makes simplifying assumptions. Probably the most significant assumption is that we cannot be sure that we have adequately addressed biases in the dataset because we do not have a ground truth for comparison [63]. Without true absence data, the model’s accuracy is heavily dependent on the number and location of background points [64]. However, not having true absence points also results in model accuracy statistics being unreliable [65]. Because the TSS data is acting as a proxy for the real underlying ecological and epidemiological processes in both the training and testing data, the model may not generalise to data gathered using a different sampling strategy (either in the UK or elsewhere) despite high performance metrics on unseen TSS data. Adding sampling study data could improve the model by providing these true absence points, particularly if under-sampled areas of geographical and environmental space were included, resulting in better model performance [66].

Further information on samplers could potentially be used to mitigate demographic differences in propensity to report vectors, and therefore further reduce bias in the model. We also recognise that population density is not necessarily indicative of human activity in popular rural destinations, and a more direct proxy for outdoor footfall would be more effective in reducing sampling bias. We have also taken at face value the point estimates from the animal density inputs, and not accounted for uncertainty in these estimates. The deer density variables were derived from previous modelling [45], and as they are very influential on the models, this is a significant limitation: less certain estimates are being given the same weight in the model as more certain estimates, for the sake of parsimony. Excluding animal density variables would remove this uncertainty, but result in much poorer predictions. Ideally, the deer estimates would be updated to use more recent underlying data, and a fully Bayesian approach used to propagate uncertainty from the deer model into the tick SDM’s posterior predictions.

Another significant limitation is that we have made many choices as researchers that have influenced the modelling process and therefore the distribution maps. For example, the choice of background point generation methods had a significant impact on the model outputs. We have aimed to blend data-first machine learning methods with some more directed methods (in particular, the GAM) to reduce our reliance on one model type, or on our priors, or on the dataset itself, but this prevented us quantifying uncertainty in as direct a manner as a Bayesian approach. We opted to ensemble the base models using a simple average across the two statistical models and the two machine learning models, despite the overall higher performance of the machine learning models. Without true absence data, we were concerned that the machine learning models’ extra flexibility to non-linear patterns implied a higher risk of overfitting to the data without generalising to unrealised parts of the species’ niche, or even to areas where the species is present but no presence records yet exist. Giving equal weight to simpler statistical models in the ensemble mitigates that risk somewhat.

For the specific case of *I. ricinus*, this model also underlines the importance of deer as a host at a time when deer are present in increasing numbers across the UK. More broadly, we hope that as public awareness of vector-borne disease increases, this will lead to better passive reporting of ticks and mosquitoes. The bias mitigation strategies applied in this study may be applicable to other passive vector surveillance datasets, enabling public health researchers to better model and understand the risks posed by these species. Better availability of risk maps for vectors has a range of potential public health benefits - these include: informing local partners’ risk reduction strategies; informing high-risk groups and outdoor space users; highlighting areas with high predicted risk, but lower numbers of actual samples for enhanced surveillance and engagement; supporting serosurveillance studies to understand how presence translates to human health risk; and aiding health care practitioners to assess patient exposure, and appropriately prioritise a differential diagnosis of vector-borne disease.

## Supporting information

S1 Text Fig A in S1 Text: Bar plot showing the total weight assigned to presences and background points, with background points broken down into random and target-group sampled subclasses. Fig B in S1 Text: Map showing the standard deviation of modelled presence probabilities across the four base models. Areas with higher standard deviation can be viewed as more uncertain, as their predictions are more affected by model design. Country boundaries source: Office for National Statistics licensed under the Open Government Licence v.3.0. Fig C in S1 Text: Panel A: Map of England and Wales showing the sparsity weighting for each area. Panel B: Histogram showing the number of MSOAs and their sparsity weightings. Country boundaries source: Office for National Statistics licensed under the Open Government Licence v.3.0. Fig D in S1 Text: Wilkinson dot plots and intervals showing the distribution of the probability assigned to points in the 2024 testing data by each base model, split by class (whether the point was a true tick presence point or a background point). Each bin represents 1% of the distribution, and each bin represents an equal number of observations (Kay, 2023). The square point shows the median, and the thick black horizontal line shows the central two quartiles of the distribution. The dashed vertical line represents the 50% threshold. Model names shown above each subplot. Fig E in S1 Text: Mean, 25th percentile and 75th percentile *I. ricinus* presence probabilities for National Parks in England and Wales. The dotted line shows the mean presence probability predicted for England and Wales overall. Fig F in S1 Text: Mean, 25th percentile and 75th percentile *I. ricinus* presence probabilities for National Landscapes (formerly Areas of Outstanding Natural Beauty) in England and Wales. The dotted line shows the mean presence probability for England and Wales overall. Fig G in S1 Text: Maps showing differences in predictions between the sensitivity testing scenarios that alter presence points and the baseline scenario. Country boundaries source: Office for National Statistics licensed under the Open Government Licence v.3.0. Fig H in S1 Text: Maps showing differences in predictions between the sensitivity testing scenarios that alter background points and the baseline scenario. Country boundaries source: Office for National Statistics licensed under the Open Government Licence v.3.0. Fig I in S1 Text: Density plot showing differences in predictions between all sensitivity testing scenarios and the baseline scenario. Fig J in S1 Text: Average Local Effect plots (ALEs) showing local predictions from the simple ensemble model, for the full range of the ten most important predictors in the testing data. Other predictors are set to locally relevant values. Table A in S1 Text: Unweighted summary statistics for variables in the training set (2013–2023) of the Tick Surveillance Scheme. Minimum (“Min”), maximum (“Max”) and mean are calculated within each class. “BG” denotes background points, while “PR” denotes presence points. Table B in S1 Text: Spearman rank correlation and mean absolute difference between each scenario and the baseline. Table C in S1 Text: Model performance metrics for the different sensitivity analysis scenarios. Table D in S1 Text: Predictive performance metrics for the alternative model ensembles. These metrics are based on the testing set, which is reports from 2024. All metrics are based on unweighted data.
(DOCX)
