# Supplementary material for: Modelling the distribution of the tick Ixodes ricinus in England and Wales using passive surveillance data from citizen science reports
Source: PLoS Negl Trop Dis. 2025 Oct 9;19(10):e0013520. doi: 10.1371/journal.pntd.0013520 (PMC12533967; doi:10.1371/journal.pntd.0013520)
Supplement: S1 Text — Fig A in S1 Text: Bar plot showing the total weight assigned to presences and background points, with background points broken down into random and target-group sampled subclasses. Fig B in S1 Text: Map showing the standard deviation of modelled presence probabilities across the four base models. Areas with higher standard deviation can be viewed as more uncertain, as their predictions are more affected by model design. Country boundaries source: Office for National Statistics licensed under the Open Government Licence v.3.0. Fig C in S1 Text: Panel A: Map of England and Wales showing the sparsity weighting for each area. Panel B: Histogram showing the number of MSOAs and their sparsity weightings. Country boundaries source: Office for National Statistics licensed under the Open Government Licence v.3.0. Fig D in S1 Text: Wilkinson dot plots and intervals showing the distribution of the probability assigned to points in the 2024 testing data by each base model, split by class (whether the point was a true tick presence point or a background point). Each bin represents 1% of the distribution, and each bin represents an equal number of observations (Kay, 2023). The square point shows the median, and the thick black horizontal line shows the central two quartiles of the distribution. The dashed vertical line represents the 50% threshold. Model names shown above each subplot. Fig E in S1 Text: Mean, 25th percentile and 75th percentile I. ricinus presence probabilities for National Parks in England and Wales. The dotted line shows the mean presence probability predicted for England and Wales overall. Fig F in S1 Text: Mean, 25th percentile and 75th percentile I. ricinus presence probabilities for National Landscapes (formerly Areas of Outstanding Natural Beauty) in England and Wales. The dotted line shows the mean presence probability for England and Wales overall. Fig G in S1 Text: Maps showing differences in predictions between the sensitivity testing scenarios that al [file pntd.0013520.s001.docx]

Modelling the distribution of the tick *Ixodes ricinus* in England and Wales using passive surveillance data from citizen science reports: Supplementary Materials

# Supplementary Materials

### Variables

Table A shows summary statistics for the training data, split into presences (PR) and background (BG) points. All statistics are unweighted.

| Table A: Unweighted summary statistics for variables in the training set (2013-2023) of the Tick Surveillance Scheme. Minimum (“Min”), maximum (“Max”) and mean are calculated within each class. “BG” denotes background points, while “PR” denotes presence points.   \|  \| **Min** \| \| **Max** \| \| **Mean** \| \| \| --- \| --- \| --- \| --- \| --- \| --- \| --- \| \| **Variable** \| **BG** \| **PR** \| **BG** \| **PR** \| **BG** \| **PR** \| \| Ground frost days: minimum \| 0.00 \| 0.00 \| 2.25 \| 2.56 \| 0.07 \| 0.08 \| \| Ground frost days: maximum * \| 0.96 \| 2.27 \| 31.00 \| 30.77 \| 18.22 \| 18.12 \| \| Rainfall (mm): minimum \| 0.00 \| 0.08 \| 133.24 \| 146.45 \| 16.55 \| 15.72 \| \| Rainfall (mm): maximum \| 58.45 \| 57.56 \| 1181.59 \| 1192.61 \| 154.04 \| 166.34 \| \| Rainfall (mm): mean * \| 32.36 \| 35.61 \| 425.41 \| 354.22 \| 75.57 \| 79.23 \| \| Highest average air temperature (°c): maximum \| 13.38 \| 13.25 \| 28.08 \| 27.84 \| 22.53 \| 22.86 \| \| Humidity (%): minimum \| 57.41 \| 58.46 \| 82.80 \| 82.85 \| 72.29 \| 71.93 \| \| Humidity (%): maximum \| 83.51 \| 84.71 \| 96.08 \| 96.26 \| 89.93 \| 89.71 \| \| Sunshine hours: minimum * \| 9.69 \| 10.87 \| 84.14 \| 79.37 \| 41.57 \| 42.27 \| \| Sunshine hours: maximum * \| 158.22 \| 167.56 \| 366.94 \| 382.88 \| 248.14 \| 254.94 \| \| Sunshine hours: mean \| 88.51 \| 91.76 \| 182.08 \| 189.55 \| 133.28 \| 136.19 \| \| Soil type: arenosol \| 0.00 \| 0.00 \| 1.00 \| 1.00 \| 0.01 \| 0.01 \| \| Soil type: cambisol \| 0.00 \| 0.00 \| 1.00 \| 1.00 \| 0.30 \| 0.24 \| \| Soil type: fluvisol \| 0.00 \| 0.00 \| 1.00 \| 1.00 \| 0.05 \| 0.02 \| \| Soil type: gleysol \| 0.00 \| 0.00 \| 1.00 \| 1.00 \| 0.12 \| 0.06 \| \| Soil type: histosol \| 0.00 \| 0.00 \| 1.00 \| 1.00 \| 0.02 \| 0.01 \| \| Soil type: leptosol \| 0.00 \| 0.00 \| 1.00 \| 1.00 \| 0.05 \| 0.09 \| \| Soil type: luvisol \| 0.00 \| 0.00 \| 1.00 \| 1.00 \| 0.34 \| 0.35 \| \| Soil type: no information \| 0.00 \| 0.00 \| 1.00 \| 1.00 \| 0.01 \| 0.01 \| \| Soil type: podzol \| 0.00 \| 0.00 \| 1.00 \| 1.00 \| 0.02 \| 0.08 \| \| Soil type: regosol \| 0.00 \| 0.00 \| 1.00 \| 1.00 \| 0.00 \| 0.00 \| \| Soil type: town \| 0.00 \| 0.00 \| 1.00 \| 1.00 \| 0.08 \| 0.13 \| \| Soil type: water body \| 0.00 \| 0.00 \| 0.56 \| 0.56 \| 0.00 \| 0.00 \| \| Geology: alluvium \| 0.00 \| 0.00 \| 1.00 \| 1.00 \| 0.10 \| 0.07 \| \| Geology: blown sand \| 0.00 \| 0.00 \| 1.00 \| 1.00 \| 0.01 \| 0.00 \| \| Geology: brickearth \| 0.00 \| 0.00 \| 1.00 \| 1.00 \| 0.01 \| 0.01 \| \| Geology: clay with flints formation \| 0.00 \| 0.00 \| 1.00 \| 1.00 \| 0.01 \| 0.03 \| \| Geology: crag group \| 0.00 \| 0.00 \| 1.00 \| 1.00 \| 0.00 \| 0.00 \| \| Geology: drift geology not mapped \| 0.00 \| 0.00 \| 1.00 \| 1.00 \| 0.00 \| 0.00 \| \| Geology: glacial sand and gravel \| 0.00 \| 0.00 \| 1.00 \| 1.00 \| 0.04 \| 0.02 \| \| Geology: lacustrine deposits \| 0.00 \| 0.00 \| 1.00 \| 1.00 \| 0.01 \| 0.00 \| \| Geology: landslide deposits \| 0.00 \| 0.00 \| 1.00 \| 1.00 \| 0.00 \| 0.01 \| \| Geology: missing \| 0.00 \| 0.00 \| 1.00 \| 1.00 \| 0.51 \| 0.64 \| \| Geology: peat \| 0.00 \| 0.00 \| 1.00 \| 1.00 \| 0.02 \| 0.01 \| \| Geology: raised marine and coastal zone deposits \| 0.00 \| 0.00 \| 1.00 \| 1.00 \| 0.00 \| 0.00 \| \| Geology: river terrace deposits \| 0.00 \| 0.00 \| 1.00 \| 1.00 \| 0.06 \| 0.08 \| \| Geology: sand and gravel of uncertain age and origin \| 0.00 \| 0.00 \| 1.00 \| 1.00 \| 0.00 \| 0.04 \| \| Geology: till \| 0.00 \| 0.00 \| 1.00 \| 1.00 \| 0.22 \| 0.08 \| \| Land cover: arable * \| 0.00 \| 0.00 \| 1.00 \| 1.00 \| 0.32 \| 0.14 \| \| Land cover: broadleaf woodland * \| 0.00 \| 0.00 \| 1.00 \| 1.00 \| 0.05 \| 0.12 \| \| Land cover: built up areas and gardens * \| 0.00 \| 0.00 \| 1.00 \| 1.00 \| 0.17 \| 0.33 \| \| Land cover: coastal \| 0.00 \| 0.00 \| 1.00 \| 1.00 \| 0.01 \| 0.01 \| \| Land cover: coniferous woodland * \| 0.00 \| 0.00 \| 1.00 \| 1.00 \| 0.02 \| 0.03 \| \| Land cover: freshwater \| 0.00 \| 0.00 \| 1.00 \| 0.99 \| 0.01 \| 0.00 \| \| Land cover: improved grassland \| 0.00 \| 0.00 \| 1.00 \| 1.00 \| 0.31 \| 0.29 \| \| Land cover: mountain, heath and bog * \| 0.00 \| 0.00 \| 1.00 \| 1.00 \| 0.03 \| 0.03 \| \| Land cover: saltwater \| 0.00 \| 0.00 \| 1.00 \| 0.87 \| 0.01 \| 0.00 \| \| Land cover: semi natural grassland \| 0.00 \| 0.00 \| 1.00 \| 1.00 \| 0.07 \| 0.04 \| \| Chinese water deer current distribution * \| 0.00 \| 0.00 \| 1.00 \| 1.00 \| 0.15 \| 0.09 \| \| Fallow deer current distribution \| 0.00 \| 0.00 \| 1.00 \| 1.00 \| 0.64 \| 0.75 \| \| Muntjac deer current distribution \| 0.00 \| 0.00 \| 1.00 \| 1.00 \| 0.71 \| 0.76 \| \| Red deer current distribution * \| 0.00 \| 0.00 \| 1.00 \| 1.00 \| 0.34 \| 0.54 \| \| Roe deer current distribution * \| 0.00 \| 0.00 \| 1.00 \| 1.00 \| 0.64 \| 0.84 \| \| Sika deer current distribution * \| 0.00 \| 0.00 \| 1.00 \| 1.00 \| 0.35 \| 0.55 \| \| Chinese water deer suitability \| 0.00 \| 0.00 \| 0.93 \| 0.93 \| 0.15 \| 0.14 \| \| Fallow deer suitability \| 0.00 \| 0.00 \| 0.93 \| 0.93 \| 0.18 \| 0.20 \| \| Muntjac deer suitability \| 0.00 \| 0.00 \| 0.97 \| 0.97 \| 0.26 \| 0.21 \| \| Red deer suitability \| 0.00 \| 0.00 \| 0.97 \| 0.97 \| 0.16 \| 0.14 \| \| Sika deer suitability \| 0.00 \| 0.00 \| 0.92 \| 0.89 \| 0.06 \| 0.07 \| \| Cattle population \| 0.04 \| 0.05 \| 181.57 \| 161.36 \| 34.28 \| 30.90 \| \| Pig population (per km^2^) \| 0.01 \| 0.01 \| 614.07 \| 402.37 \| 25.69 \| 15.49 \| \| Sheep population * \| 0.10 \| 0.14 \| 442.58 \| 399.90 \| 62.96 \| 46.92 \| \| Elevation: standard deviation \| 0.00 \| 0.00 \| 142.00 \| 128.26 \| 11.37 \| 13.62 \| \| NDVI (Normalized Difference Vegetation Index) \| -0.04 \| 0.01 \| 0.51 \| 0.49 \| 0.34 \| 0.34 \| \| Weights \| 0.10 \| 0.06 \| 0.40 \| 1.85 \| 0.25 \| 0.99 \| \| Single asterisk (*) : variable included in generalised additive model (GAM) formula. \| \| \| \| \| \| \| |
| --- | --- | --- | --- | --- | --- | --- | --- | --- | --- | --- | --- | --- | --- | --- | --- | --- | --- | --- | --- | --- | --- | --- | --- | --- | --- | --- | --- | --- | --- | --- | --- | --- | --- | --- | --- | --- | --- | --- | --- | --- | --- | --- | --- | --- | --- | --- | --- | --- | --- | --- | --- | --- | --- | --- | --- | --- | --- | --- | --- | --- | --- | --- | --- | --- | --- | --- | --- | --- | --- | --- | --- | --- | --- | --- | --- | --- | --- | --- | --- | --- | --- | --- | --- | --- | --- | --- | --- | --- | --- | --- | --- | --- | --- | --- | --- | --- | --- | --- | --- | --- | --- | --- | --- | --- | --- | --- | --- | --- | --- | --- | --- | --- | --- | --- | --- | --- | --- | --- | --- | --- | --- | --- | --- | --- | --- | --- | --- | --- | --- | --- | --- | --- | --- | --- | --- | --- | --- | --- | --- | --- | --- | --- | --- | --- | --- | --- | --- | --- | --- | --- | --- | --- | --- | --- | --- | --- | --- | --- | --- | --- | --- | --- | --- | --- | --- | --- | --- | --- | --- | --- | --- | --- | --- | --- | --- | --- | --- | --- | --- | --- | --- | --- | --- | --- | --- | --- | --- | --- | --- | --- | --- | --- | --- | --- | --- | --- | --- | --- | --- | --- | --- | --- | --- | --- | --- | --- | --- | --- | --- | --- | --- | --- | --- | --- | --- | --- | --- | --- | --- | --- | --- | --- | --- | --- | --- | --- | --- | --- | --- | --- | --- | --- | --- | --- | --- | --- | --- | --- | --- | --- | --- | --- | --- | --- | --- | --- | --- | --- | --- | --- | --- | --- | --- | --- | --- | --- | --- | --- | --- | --- | --- | --- | --- | --- | --- | --- | --- | --- | --- | --- | --- | --- | --- | --- | --- | --- | --- | --- | --- | --- | --- | --- | --- | --- | --- | --- | --- | --- | --- | --- | --- | --- | --- | --- | --- | --- | --- | --- | --- | --- | --- | --- | --- | --- | --- | --- | --- | --- | --- | --- | --- | --- | --- | --- | --- | --- | --- | --- | --- | --- | --- | --- | --- | --- | --- | --- | --- | --- | --- | --- | --- | --- | --- | --- | --- | --- | --- | --- | --- | --- | --- | --- | --- | --- | --- | --- | --- | --- | --- | --- | --- | --- | --- | --- | --- | --- | --- | --- | --- | --- | --- | --- | --- | --- | --- | --- | --- | --- | --- | --- | --- | --- | --- | --- | --- | --- | --- | --- | --- | --- | --- | --- | --- | --- | --- | --- | --- | --- | --- | --- | --- | --- | --- | --- | --- | --- | --- | --- | --- | --- | --- | --- | --- | --- | --- | --- | --- | --- | --- | --- | --- | --- | --- | --- | --- | --- | --- | --- | --- | --- | --- | --- | --- | --- | --- | --- | --- | --- | --- | --- | --- | --- | --- | --- | --- | --- | --- | --- | --- | --- | --- | --- | --- | --- | --- | --- | --- | --- | --- | --- | --- | --- | --- | --- | --- | --- | --- | --- | --- | --- | --- | --- | --- | --- | --- | --- | --- | --- | --- | --- | --- | --- | --- | --- | --- | --- |

### Background point weighting

Figure A demonstrates the difference in overall weighting between presence points and the two different types of background point.

| 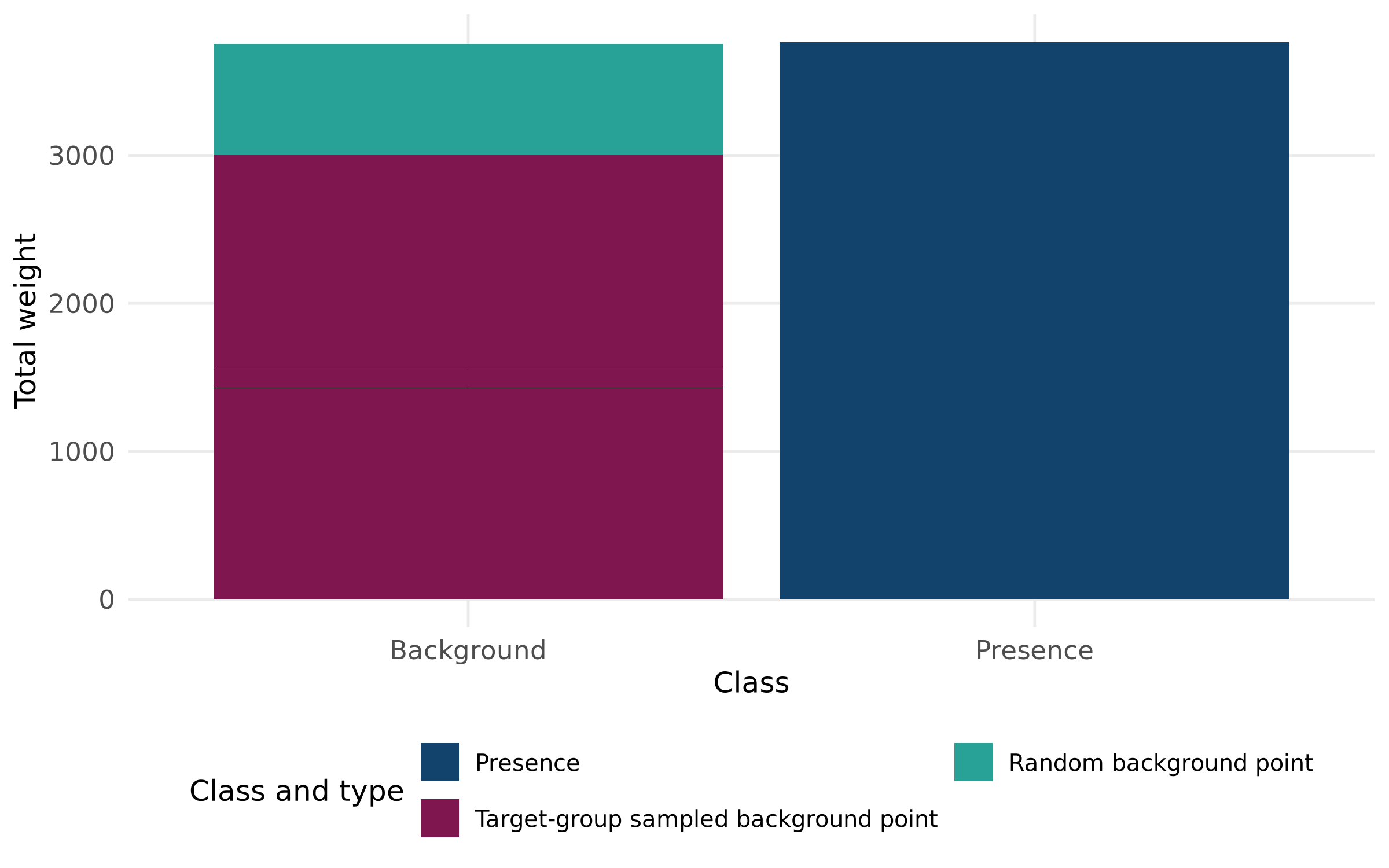  Figure A: Bar plot showing the total weight assigned to presences and background points, with background points broken down into random and target-group sampled subclasses. |
| --- |

### Uncertainty

Figure B gives an idea of uncertainty by visualising the standard deviation of modelled presence probabilities across the four base models (penalised GLM, GAM, XGBoost and Random Forest).

| 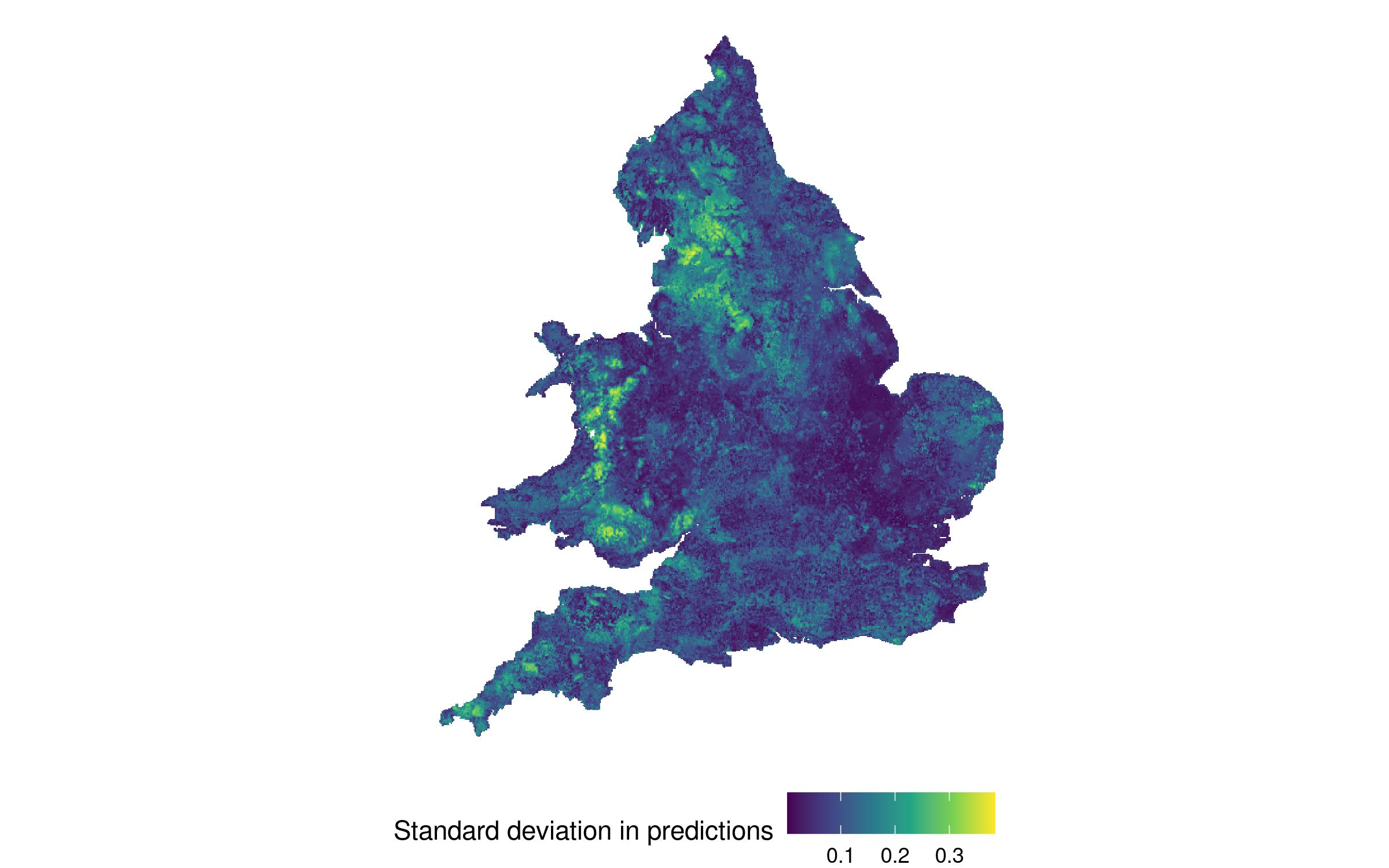  Figure B: Map showing the standard deviation of modelled presence probabilities across the four base models. Areas with higher standard deviation can be viewed as more uncertain, as their predictions are more affected by model design. Country boundaries source: Office for National Statistics licensed under the Open Government Licence v.3.0. |
| --- |

The areas where predictions vary most between model are in the North West of England and in parts of Wales. These areas are likely to be relatively more suitable according to the simpler linear statistical regressions than the more complex patterns modelled by the machine learning models; as Figure D showed, XGBoost and Random Forest assigned low probabilities more often than the pGLM and GAM.

### Model hyperparameters

In the final selected penalized GLM model, alpha (the mixture argument) was set to 0.471 and lambda (the penalty argument) was set to 0. The pGLM was therefore an elastic net model (Zhou and Hastie 2005). The variables rainfall (mm): minimum, sunshine hours: maximum *, soil type: cambisol and geology: missing were removed by the penalization.

For the XGBoost model, the number of trees was set at 1000, with early stopping enabled to allow a smaller number of trees to be generated as required. The learning rate was set at 0.01. The proportion of variables included in training, the maximum tree depth, the minimum loss reduction and the number of early stopping iterations are all set by tuning as described earlier. The single best hyperparameter combination in the main xgBoost specification used all variables, a maximum tree depth of 8, a minimum loss reduction of 0.0004288 and 17 early stopping rounds.

For the Random Forest model, hyperparameter tuning determined the number of trees, the number of predictors sampled for each split, and the minimum node size. In the best version of the Random Forests model, 198 trees are used, with 46 predictors randomly sampled for each split, and a minimum of 10 datapoints is required to split a node further.

### Population sparsity

Figure C shows the population sparsity weighting applied to each Middle layer Super Output Area (MSOA). The histogram shows how many MSOAs had sparsity weights within 1% bins.

| 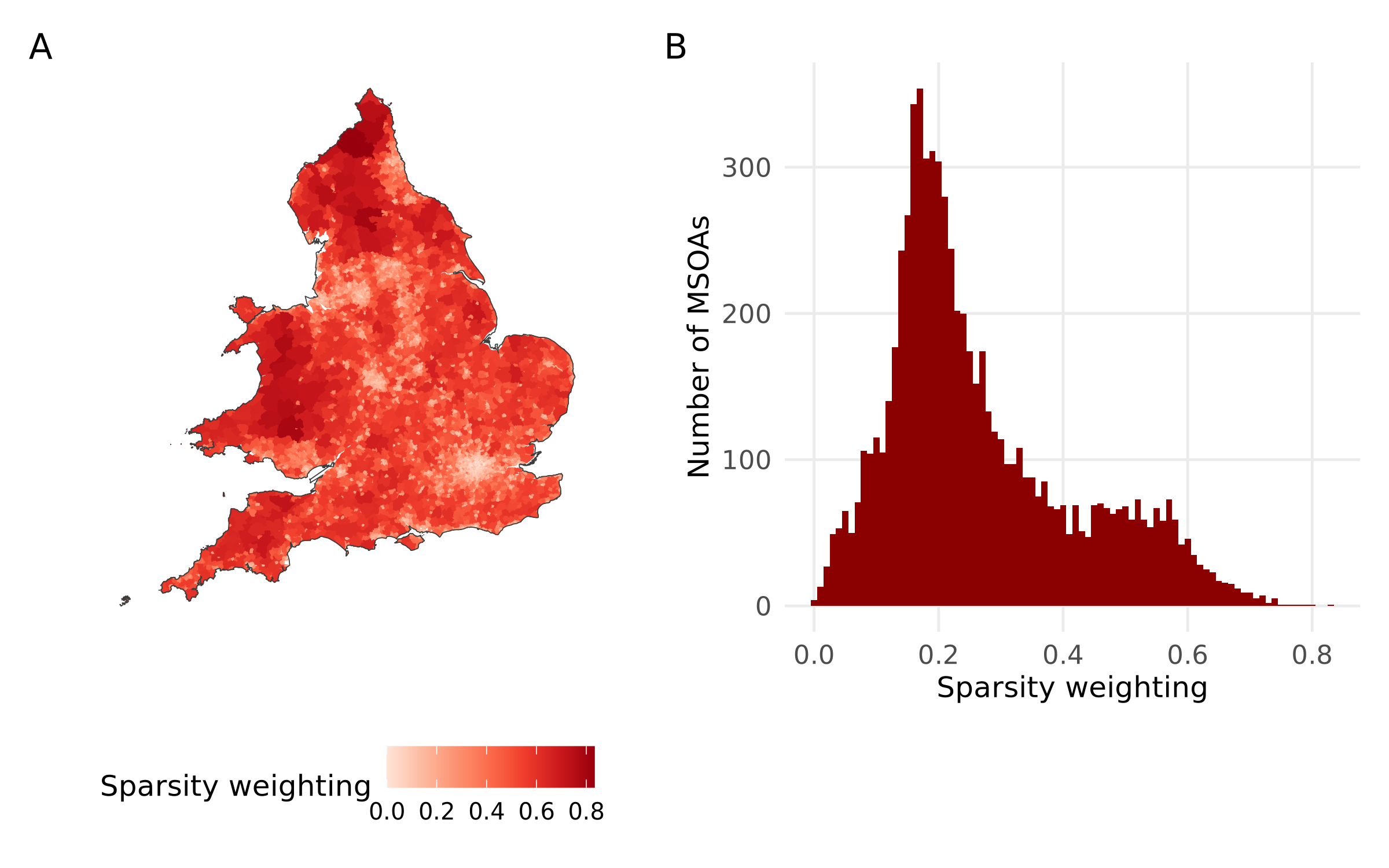  Figure C: Panel A: Map of England and Wales showing the sparsity weighting for each area. Panel B: Histogram showing the number of MSOAs and their sparsity weightings. Country boundaries source: Office for National Statistics licensed under the Open Government Licence v.3.0. |
| --- |

### Base model predictions

Figure D replicates the Wilkinson dotplot from the main Results section for each of the ensemble’s base models.

| 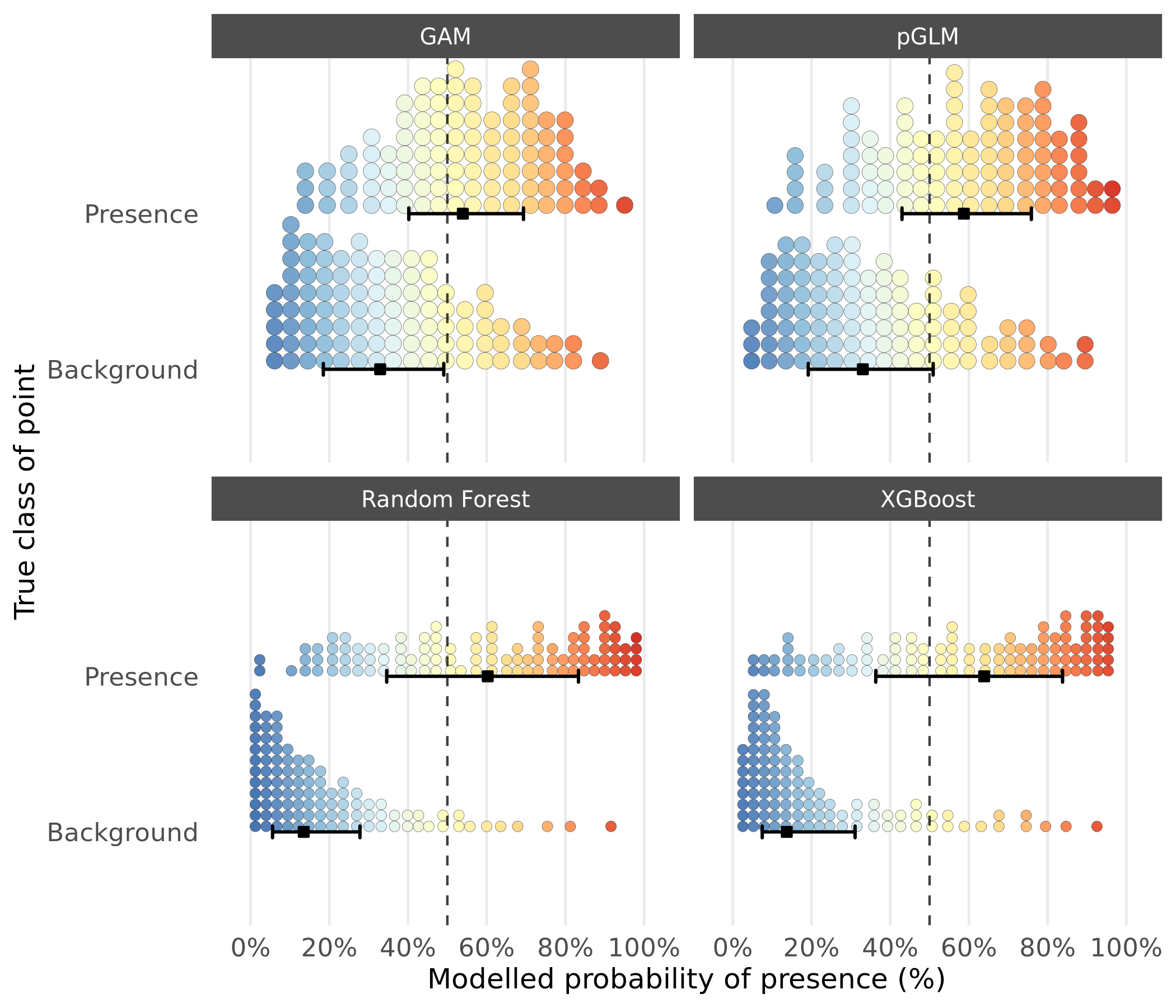  Figure D: Wilkinson dot plots and intervals showing the distribution of the probability assigned to points in the 2024 testing data by each base model, split by class (whether the point was a true tick presence point or a background point). Each bin represents 1% of the distribution, and each bin represents an equal number of observations (Kay, 2023). The square point shows the median, and the thick black horizontal line shows the central two quartiles of the distribution. The dashed vertical line represents the 50% threshold. Model names shown above each subplot. |
| --- |

In the 2024 testing data, the machine learning models were more confident and accurate in assigning background points correctly: for example, the Random Forest model gave a median prediction of 14% to background points. This likely reflects the flexible non-linear nature of tree-based machine learning methods, which can effectively account for different relationships between variables in distinct habitats or regions.

### National Parks and National Landscapes

Looking at public spaces with the highest probability of *I. ricinus* presence, the New Forest, Lake District and Exmoor are the National Parks had the highest predictions, while the Brecon Beacons, Pembrokeshire Coast and the Norfolk and Suffolk Broads had the lowest. For the National Landscapes, the Surrey Hills, Arnside & Silverdale and East Devon have high predictions, while the Lincolnshire Wolds, Wye Valley and Cannock Chase have with the lowest predictions

Recognised tick and Lyme hotspots in the medical literature such as the New Forest, Exmoor, the South Downs, the Lake District and the North Yorkshire Moors [1] are all assigned much higher presence probabilities than average; see Figure E for a comparison of National Parks, and Figure F for a comparison of National Landscapes (formerly Areas of Outstanding Natural Beauty).

| 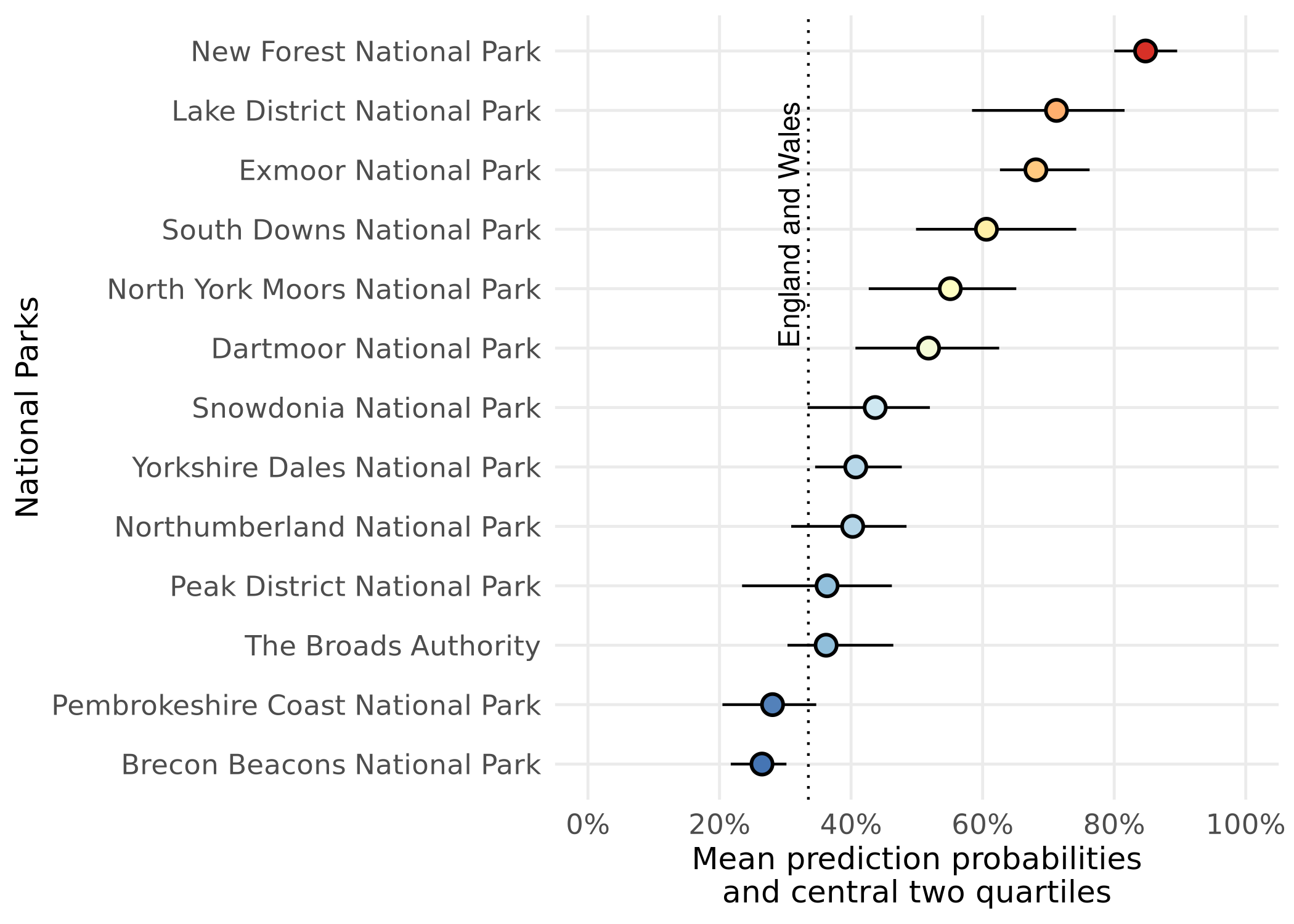  Figure E: Mean, 25th percentile and 75th percentile I. ricinus presence probabilities for National Parks in England and Wales. The dotted line shows the mean presence probability predicted for England and Wales overall. |
| --- |
| 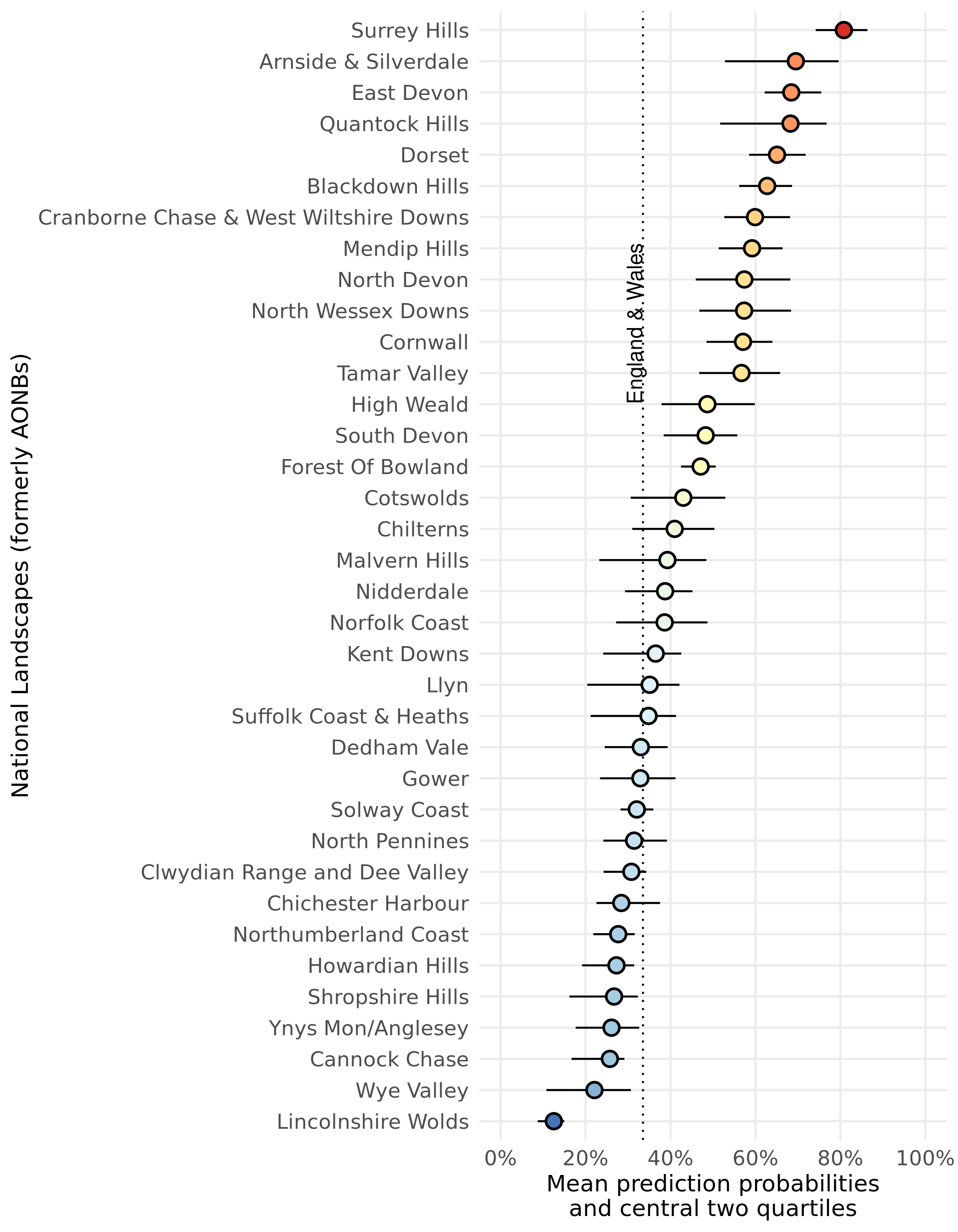  Figure F: Mean, 25th percentile and 75th percentile I. ricinus presence probabilities for National Landscapes (formerly Areas of Outstanding Natural Beauty) in England and Wales. The dotted line shows the mean presence probability for England and Wales overall. |

By contrast, heavily urban areas such as central London are not designated as hotspots, with the exception of extensive green spaces like Richmond Park where ticks and deer are known to be present [1,2]. The mean presence prediction in the Lower layer Super Output Areas (LSOAs) that cover most of Richmond Park (Richmond upon Thames 012B, 012C and 012 in the 2021 LSOA boundaries) is 63%. This compares to the median LSOA’s presence probability of 29%. Lower layer Super Output Areas (2021) Boundaries EW BSC were taken from the ONS Open Geography Portal [3].

### Sensitivity Testing

Three scenarios are tested that alter the presence points: in the first, presence points have spatial thinning applied with a minimum distance of 10km; in the second, no population weighting is applied; and in the third, only human records are used. To facilitate visual comparison between similar prediction maps, Figure G shows the binned difference in predictions between each scenario and the baseline.

| 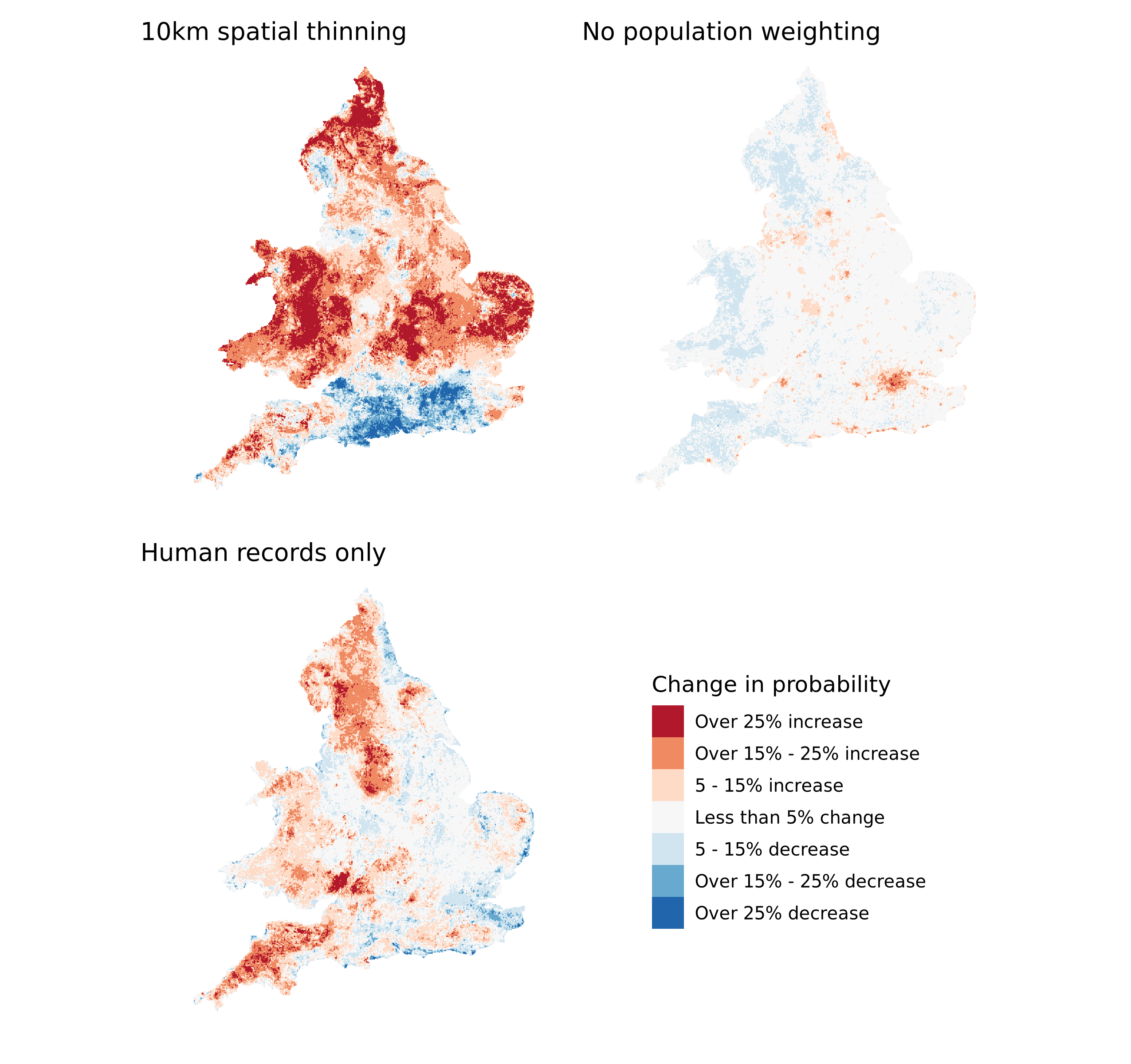  Figure G: Maps showing differences in predictions between the sensitivity testing scenarios that alter presence points and the baseline scenario. Country boundaries source: Office for National Statistics licensed under the Open Government Licence v.3.0. |
| --- |

As Figure G demonstrates, introducing spatial thinning at 10km distance has considerable effects on the model predictions in both directions across most of the study area. Removing population weighting, as expected, increases the probability of presence in urban centres; filtering the data to human only increases the probability of presence in south-east Wales, Cornwall, and in much of the North West and Cumbria (note that some of this effect will be due to the reduction in overall numbers of presences).

A further five scenarios altered how background points were generated, and are shown in Figure H. In the first, only random background points are used to train the model; in the second, random and target-group sampled points are equally weighted, and in the third only target-group sampled points are used to train the model. The fourth scenario increases the ratio of background to presence points from 4:1to 10:1. Finally, the fifth scenario removes the distance limit between presence points and background points (which is 5km in the baseline scenario).

| 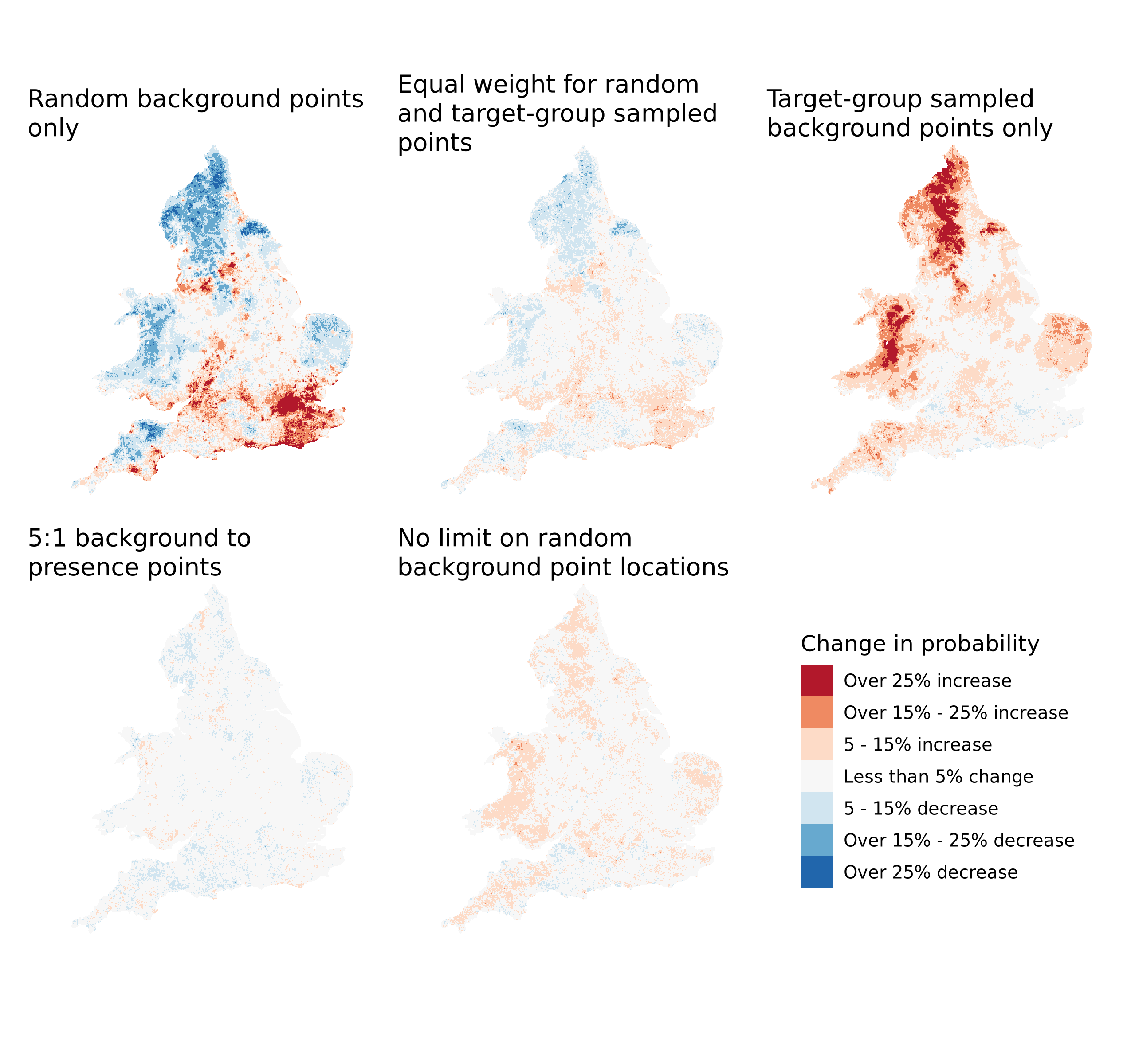  Figure H: Maps showing differences in predictions between the sensitivity testing scenarios that alter background points and the baseline scenario. Country boundaries source: Office for National Statistics licensed under the Open Government Licence v.3.0. |
| --- |

Figure H shows that increasing numbers of background points or removing the limit on how close random background points can be to presence points makes only minor differences to the predictions. Changes in the proportion of target-group sampled and random background points have significant effects on Greater London and the South, as well as Wales, the North West and the North York Moors. This suggests that the target-group sampling strategy is effective in putting emphasis on rural environments and addressing potential pro-urban spatial bias.

Figure I shows the overall distribution of changes from the baseline scenario. To quantify differences between predictions, Table B shows the Spearman rank correlation and mean absolute difference between each scenario and the baseline. The spatial thinning scenario has the highest mean absolute difference and lowest Spearman rank correlation.

| 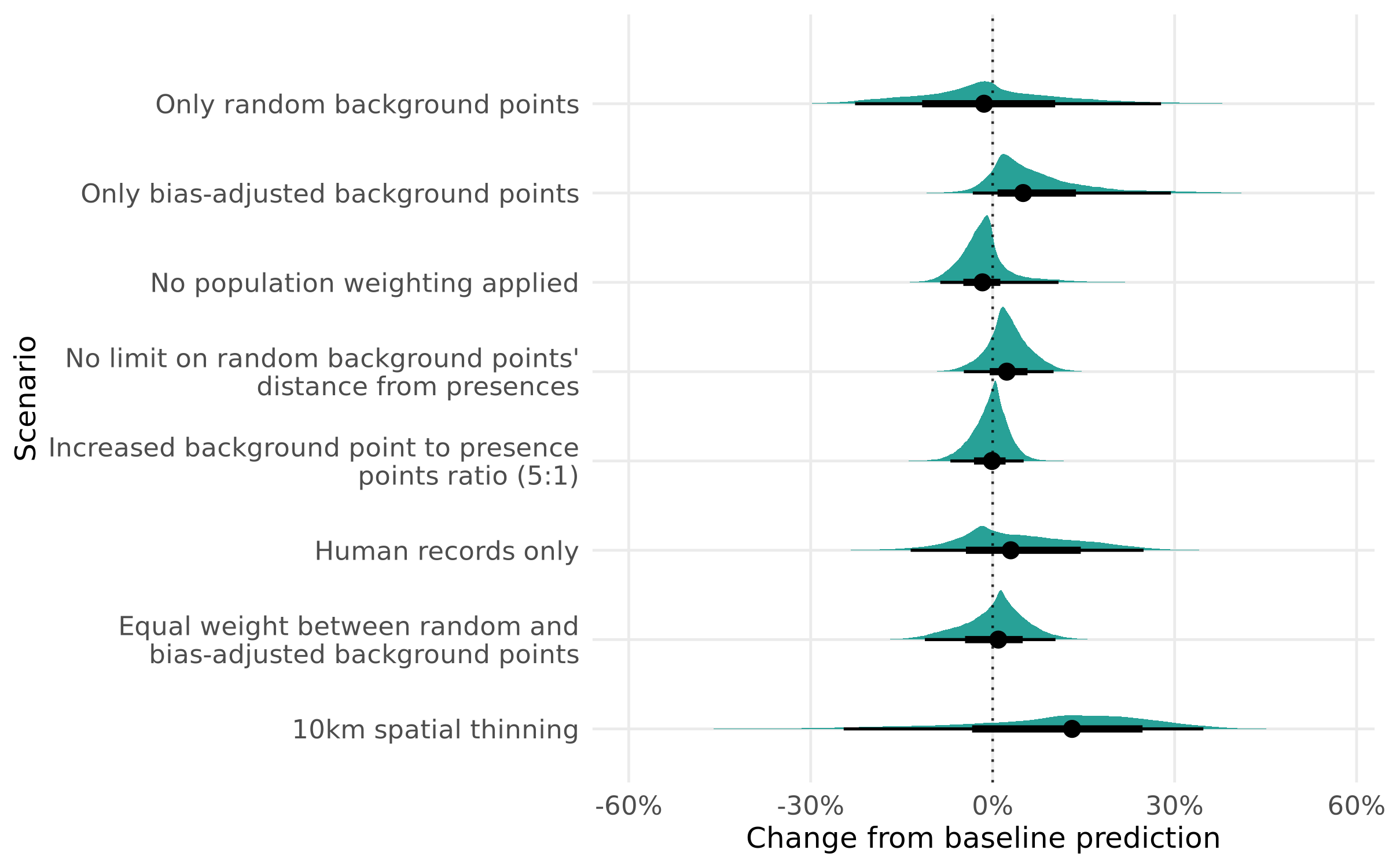  Figure I: Density plot showing differences in predictions between all sensitivity testing scenarios and the baseline scenario. |
| --- |
| Table B: Spearman rank correlation and mean absolute difference between each scenario and the baseline.   \| **Scenario** \| **Spearman correlation** \| **Mean absolute difference** \| \| --- \| --- \| --- \| \| Human records only \| 0.92 \| 0.08 \| \| 10km spatial thinning \| 0.71 \| 0.16 \| \| Increased background point to presence points ratio (5:1) \| 0.99 \| 0.02 \| \| No limit on random background points' distance from presences \| 0.99 \| 0.03 \| \| Only random background points \| 0.83 \| 0.09 \| \| Only bias-adjusted background points \| 0.95 \| 0.08 \| \| Equal weight between random and bias-adjusted background points \| 0.96 \| 0.04 \| \| No population weighting applied \| 0.97 \| 0.03 \| |

As a final check on how the scenarios described in the main text of the paper differ, Table C shows how the success metrics vary by scenario. Note that these are not fully comparable due to differences in background points. All scenarios except “Only random background points” are tested against both bias adjusted and random points, without weighting applied; that scenario is tested only against random background points, to make for a fairer comparison.

| Table C: Model performance metrics for the different sensitivity analysis scenarios.   \|  \| **Class predictions** \| \| \| \| \| **Probability predictions** \| \| \| \| \| --- \| --- \| --- \| --- \| --- \| --- \| --- \| --- \| --- \| --- \| \| **Scenario** \| **Accuracy** \| **Kappa** \| **MCC** \| **Sensitivity** \| **Specificity** \| **Boyce Continuous** \| **ROC AUC** \| **Brier score** \| **Max TSS** \| \| Baseline \| 0.81 \| 0.46 \| 0.46 \| 0.62 \| 0.86 \| 0.99 \| 0.84 \| 0.14 \| 0.54 \| \| Human records only \| 0.82 \| 0.51 \| 0.52 \| 0.74 \| 0.84 \| 0.97 \| 0.86 \| 0.14 \| 0.61 \| \| 10km spatial thinning \| 0.68 \| 0.11 \| 0.11 \| 0.36 \| 0.77 \| 0.66 \| 0.60 \| 0.21 \| 0.19 \| \| Increased background point to presence points ratio (5:1) \| 0.84 \| 0.33 \| 0.35 \| 0.60 \| 0.86 \| 0.99 \| 0.83 \| 0.13 \| 0.51 \| \| No limit on random background points' distance from presences \| 0.78 \| 0.40 \| 0.40 \| 0.62 \| 0.82 \| 0.98 \| 0.82 \| 0.15 \| 0.52 \| \| Only random background points \| 0.77 \| 0.49 \| 0.49 \| 0.67 \| 0.82 \| 0.95 \| 0.85 \| 0.15 \| 0.54 \| \| Only bias-adjusted background points \| 0.80 \| 0.56 \| 0.56 \| 0.69 \| 0.86 \| 0.98 \| 0.85 \| 0.15 \| 0.58 \| \| Equal weight between random and bias-adjusted background points \| 0.80 \| 0.43 \| 0.44 \| 0.63 \| 0.84 \| 0.98 \| 0.83 \| 0.15 \| 0.54 \| \| No population weighting applied \| 0.82 \| 0.47 \| 0.47 \| 0.63 \| 0.86 \| 0.99 \| 0.84 \| 0.14 \| 0.53 \| |
| --- | --- | --- | --- | --- | --- | --- | --- | --- | --- | --- | --- | --- | --- | --- | --- | --- | --- | --- | --- | --- | --- | --- | --- | --- | --- | --- | --- | --- | --- | --- | --- | --- | --- | --- | --- | --- | --- | --- | --- | --- | --- | --- | --- | --- | --- | --- | --- | --- | --- | --- | --- | --- | --- | --- | --- | --- | --- | --- | --- | --- | --- | --- | --- | --- | --- | --- | --- | --- | --- | --- | --- | --- | --- | --- | --- | --- | --- | --- | --- | --- | --- | --- | --- | --- | --- | --- | --- | --- | --- | --- | --- | --- | --- | --- | --- | --- | --- | --- | --- | --- | --- | --- | --- | --- | --- | --- | --- | --- | --- | --- |

In addition to taking a simple average of predictions (the ‘simple ensemble’ strategy), we also tested putting more weight on some models than others. We took weighted averages of the predictions from the best iterations of the individual base models, both using metric targeting and manual choice. To target performance metrics, we took the sum of the ROC AUC and maximum True Skill Score metrics on the training data separately (NB: our preferred overall performance metric is the continuous Boyce index, but there was little variation between the models on this metric in the training set). We then weighted each model proportionally to its contribution to the summed metric across the four models; this aims to give more weight to models that give better performance. We also manually specified two weighting schemes to illustrate the impact of changing weights: a statistics-focused scheme that assigned 45% of the weight to each of the pGLM and GAM, and 5% to each of the xgBoost and Random Forest models, and a machine learning-focused scheme with the inverse weightings (45% for xgBoost and for Random Forest, 5% each for pGLM and GAM). Table D shows the performance on the testing set from the alternative model ensembling strategies, with the simple ensemble performance repeated for ease of comparison.

| Table D: Predictive performance metrics for the alternative model ensembles. These metrics are based on the testing set, which is reports from 2024. All metrics are based on unweighted data.   \| **Metric** \| **Simple ensemble** \| **ML-focused** \| **Stats-focused** \| **Max AUC** \| **Max TSS** \| \| --- \| --- \| --- \| --- \| --- \| --- \| \| Accuracy \| 0.82 \| 0.83 \| 0.74 \| 0.82 \| 0.83 \| \| Kappa \| 0.47 \| 0.49 \| 0.33 \| 0.47 \| 0.49 \| \| Sensitivity \| 0.63 \| 0.61 \| 0.61 \| 0.62 \| 0.62 \| \| Specificity \| 0.86 \| 0.88 \| 0.78 \| 0.87 \| 0.88 \| \| Boyce Continuous \| 0.99 \| 0.99 \| 0.99 \| 0.99 \| 0.99 \| \| ROC AUC \| 0.84 \| 0.86 \| 0.79 \| 0.85 \| 0.85 \| \| Brier score \| 0.14 \| 0.12 \| 0.17 \| 0.13 \| 0.13 \| \| Max TSS \| 0.53 \| 0.57 \| 0.45 \| 0.55 \| 0.56 \| |
| --- | --- | --- | --- | --- | --- | --- | --- | --- | --- | --- | --- | --- | --- | --- | --- | --- | --- | --- | --- | --- | --- | --- | --- | --- | --- | --- | --- | --- | --- | --- | --- | --- | --- | --- | --- | --- | --- | --- | --- | --- | --- | --- | --- | --- | --- | --- | --- | --- | --- | --- | --- | --- | --- | --- |

The weighted average-based ensembles did not diverge dramatically from the simple average ensemble; placing more weight on machine learning models again increased specificity at the expense of sensitivity, whereas extra weight on the GAM and pGLM resulted in higher sensitivity than any other model strategy. Attempting to maximise specific performance metrics based on the training data was unsuccessful, indicating that the differences in model performance were not generalisable to new data.

### Average Local Effect plots

Partial dependence plots do not account for interactions and may be misleading when variables are highly correlated, in which case an accumulated local effects (ALE) plot is preferred [4]. In this case, the ALE plots are very similar to the PDP plots shown in the main results.

| 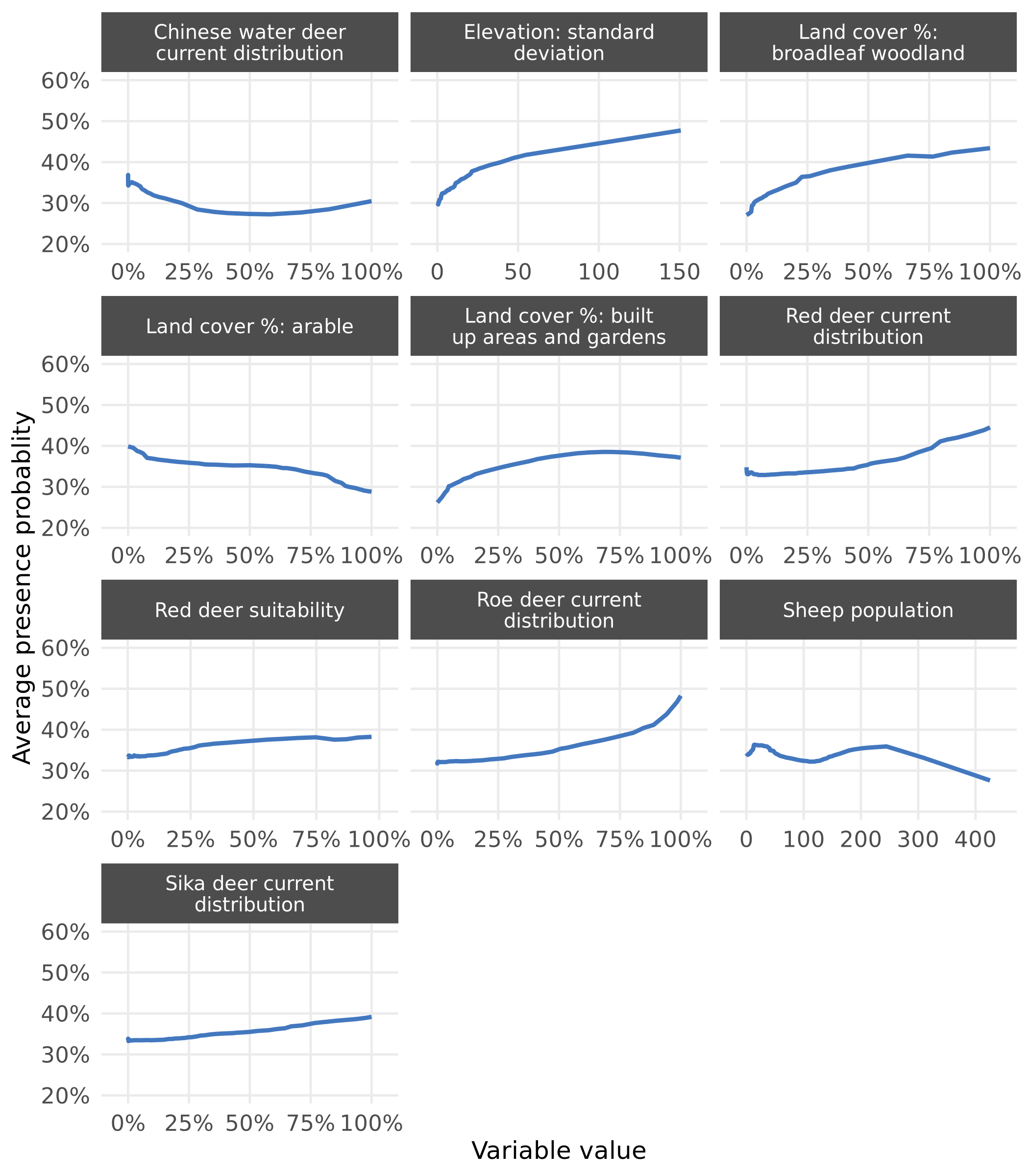  Figure J: Average Local Effect plots (ALEs) showing local predictions from the simple ensemble model, for the full range of the ten most important predictors in the testing data. Other predictors are set to locally relevant values. |
| --- |

# References (Supplementary Materials)

1. Dubrey SW, Bhatia A, Woodham S, Rakowicz W. Lyme disease in the united kingdom. Postgraduate medical journal. 2014;90: 33–42.

2. Hansford KM, McGinley L, Wilkinson S, Gillingham EL, Cull B, Gandy S, et al. Ixodes ricinus and borrelia burgdorferi sensu lato in the royal parks of london, UK. Experimental and Applied Acarology. 2021;84: 593–606.

3. Office for National Statistics. Lower layer super output areas (december 2021) boundaries EW BSC (V4). 2024. Available: <https://geoportal.statistics.gov.uk/datasets/ons::lower-layer-super-output-areas-december-2021-boundaries-ew-bsc-v4-2/about>

4. Apley DW, Zhu J. Visualizing the effects of predictor variables in black box supervised learning models. Journal of the Royal Statistical Society Series B: Statistical Methodology. 2020;82: 1059–1086.
